# Comparison of the accelerometer-based navigation system with conventional instruments for total knee arthroplasty: a propensity score-matched analysis

**DOI:** 10.1186/s13018-019-1258-y

**Published:** 2019-07-18

**Authors:** Xiang Gao, Yu Sun, Zhao-He Chen, Tian-Xu Dou, Qing-Wei Liang, Xu Li

**Affiliations:** Department of Orthopedics, The First Hospital of China Medical University, 155, Shenyang, 110000 Liaoning Province People’s Republic of China

**Keywords:** Primary total knee arthroplasty, Accelerometer-based navigation, Surgical technique, Mechanical axis, Blood loss

## Abstract

**Background:**

The accelerometer-based navigation (ABN) system is an emerging navigation system for total knee arthroplasty (TKA). This study aimed to determine whether the ABN system could improve the accuracy of mechanical alignment, component positioning, and short-term clinical outcomes for TKA when compared to conventional instruments (CON).

**Methods:**

A total of 204 patients were selected and divided into two groups (CON: 135, ABN: 69) after applying the inclusion and exclusion criteria. Then, 1:1 propensity score matching was performed for age, gender, body mass index, hip-knee-ankle angle (HKA), Knee Society Score (KSS), Western Ontario and McMaster Universities (WOMAC) score, and follow-up times. A total of 82 consecutive patients (82 knees) underwent total knee arthroplasty using ABN (*n* = 41) or CON (*n* = 41) were enrolled in this study. The postoperative HKA, frontal femoral component (FFC) angle, frontal tibial component (FTC) angle, lateral femoral flexion (LFF) angle, and lateral tibial component (LTC) angle were compared between the two groups to evaluate mechanical alignment and component positioning. Additional clinical parameters, including haemoglobin reduction, the KSS, and the WOMAC score, were assessed at the final follow-up (the mean follow-up period was 20.9 months in the CON group and 21.2 months in the ABN group).

**Results:**

The ABN group had a significantly improved mean absolute deviation of HKA (*P* = 0.033), FFC (*P* = 0.004), FTC (*P* = 0.017), LFF (*P* = 0.023), and LTC (*P* = 0.031) compared to those of the CON group. The numbers of FFCs and LTCs within 3° were significantly different (*P* = 0.021, *P* = 0.023, respectively) between the two groups. However, no differences in the numbers of FTCs within 3° (*P* = 0.166) and LFF within 3° (*P* = 0.556) were found. The ABN group had a significantly higher KS function score (*P* = 0.032), and the pain and stiffness scores were significantly different (*P* = 0.034, *P* = 0.020, respectively) between the two groups. Moreover, the ABN system could reduce hidden blood loss postoperatively. However, no difference was found in the KS knee score and the total WOMAC score between the two groups.

**Conclusion:**

This study demonstrates that ABN system improved TKA mechanical alignment and component positioning and decreased the hidden blood loss postoperatively compared to conventional instruments. However, no significant differences were found in short-term clinical outcomes between ABN and conventional instruments at the final follow-up. However, whether this system contributes to revision rates and long-term clinical outcomes requires further study.

## Background

Total knee arthroplasty (TKA) is an effective treatment to reduce pain and restore normal function and alignment in cases with advanced osteoarthritis of the knee. Ideal alignment is widely viewed as a critical factor for the long-term success of TKA [1, 2]. Nevertheless, in Parratte’s study [3], a postoperative mechanical axis of neutral ± 3° did not improve the implant survival rate after a 15-year follow-up. Although the ideal range of mechanical alignment for successful TKA is controversial, most surgeons favour mechanical alignment within 3° of neutral. Berend et al. followed 3124 TKA patients for 2–14 years and found that the failure rate increased by 17.2-fold in tibias with greater than 3° of varus and by 168-fold in tibias with greater than 3° of varus and in patients with a body mass index (BMI) > 33.7 [4]. Favourable alignment has a significant impact on function [2, 5, 6], pain [7], and quality of life [8, 9]. Ritter et al. [10] documented that among 6070 knees, only 60% achieved optimal tibial alignment (90° of valgus), femoral alignment (< 8.0° of valgus), and overall alignment (2.5–7.4° of valgus) simultaneously. Patients with severe deformities, a high BMI, and acute bowing can also limit the use of conventional instruments. Consequently, new and advanced techniques have been developed and combined with conventional instruments to assist surgeons in improving the precision of the mechanical axis and component alignment. One such innovation is the Computer Assist System (CAS). In Mason’s meta-analysis [11] of 29 studies, the percentage of femoral varus/valgus alignments within 2° perpendicular to the femoral mechanical axis was 90.4% in the CAS group (versus 65.9% in the conventional group), and the percentage of tibial varus/valgus alignment within 2° perpendicular to the tibial mechanical axis was 95.2% in the CAS group (versus 79.7% in the conventional group). However, some studies [12, 13] have noted that CAS has not been widely accepted due to inevitable problems, such as large initial start-up costs, extra pin sites, a substantial learning curve, and large consoles. Kim et al. [14] found that using CAS during TKA could lead to femoral notching and pin-site fracture. Moreover, extensive bone resection, intramedullary positioning, and soft-tissue release in TKA often contribute to significant blood loss [15]; thus, avoiding extra injury is necessary when using a navigation system.

Recently, the novel navigation system accelerometer-based navigation (ABN) has been used to avoid such negative outcomes. ABN does not require a large computer console, extra pin sites, navigation arrays, and intramedullary mechanical devices. Since the surgical procedure of ABN is similar to that of a conventional instrument, it has a shorter learning curve compared to CAS. However, there exists uncertainty whether this kind of accelerometer-based navigation can improve mechanical and component alignment or not [16, 17].

As accelerometer-based navigation systems are a relatively new technique, literatures evaluating its accuracy and clinical effectiveness in TKA are sparse. The purpose of this study is to compare the postoperative outcomes, including hip-knee-ankle angle (HKA), frontal femoral component (FFC), frontal tibial component (FTC), lateral femoral flexion (LFF), lateral tibial component (LTC), blood loss volume, Knee Society Score (KSS), and Western Ontario and McMaster Universities (WOMAC) score, in patients who underwent TKA surgery with conventional instruments and accelerometer-based navigation system. We hypothesised that using accelerometer-based navigation system during TKA would significantly improve the accuracy of mechanical alignment, component positioning, and short-term (mean follow-up period was 20.9 months in the CON group and 21.2 months in the ABN group) clinical outcomes postoperatively when compared to conventional instruments in TKA.

## Materials and methods

### Patient selection

This study was a retrospective, case-control study of patients who underwent TKA surgery with either conventional instruments or the ABN (iAssist, Zimmer, Inc., Warsaw, IN) in The First Hospital of China Medical University. The inclusion criteria were as follows: (1) a diagnosis of primary tricompartmental osteoarthritis and (2) a history of unilateral TKA after conservative treatment failure. The exclusion criteria were as follows: (1) a knee-related operative or injury history, (2) a severe knee deformity (defined as a hip-knee-ankle (HKA) angle valgus > 30° or varus > 20°), (3) rheumatoid arthritis or another medical disease involving the knee, (4) haematological disorders, and (5) hip pathology that severely limited the range of motion. A total of 204 patients were enrolled in this study after applying the inclusion and exclusion criteria. From April 2016 to November 2017, 69 patients received a TKA using the iAssist to perform distal femoral and proximal tibial resection. During the same period, 135 patients received a TKA using conventional instruments. All procedures were performed by one experienced surgeon being familiar with the iAssist navigation system. This retrospective study was approved by the Ethics Committee of The First Hospital of China Medical University.

### Propensity-matched analysis

Propensity score matching analysis are statistical methods aimed to reduce confounding attributable to measured covariates in observational studies [18]. In observational studies, there is often bias derived from significant differences between the characteristic subjects of the treatment group and the no-treatment group. We attempted to limit such bias with propensity score-matched analysis to account for other possible confounding factors. A propensity score is generally defined as a patient’s conditional probability of being assigned a treatment based on patient’s pre-treatment characteristics by logistic regression using the “MatchIt” R package. Then, the ABN and CON groups were propensity score matched in a 1:1 ratio based on age, gender, BMI, HKA angel, Knee Society Score (KSS), and Western Ontario and McMaster Universities (WOMAC) score.

### Surgical technique for the ABN group

The iAssist system is a novel, personalised guide system that is designed to guide both proximal tibial and distal femoral resections in TKA. The system uses surgical instruments and positioning sensors to determine the axes in relation to anatomical landmarks. The surgical workflow is similar to the conventional method, with independent resection of the femur and tibia along their respective mechanical axes. The only difference of surgical procedure between the two groups is the tool used to guide both proximal tibial and distal femoral resections.

**Fig. 1:**
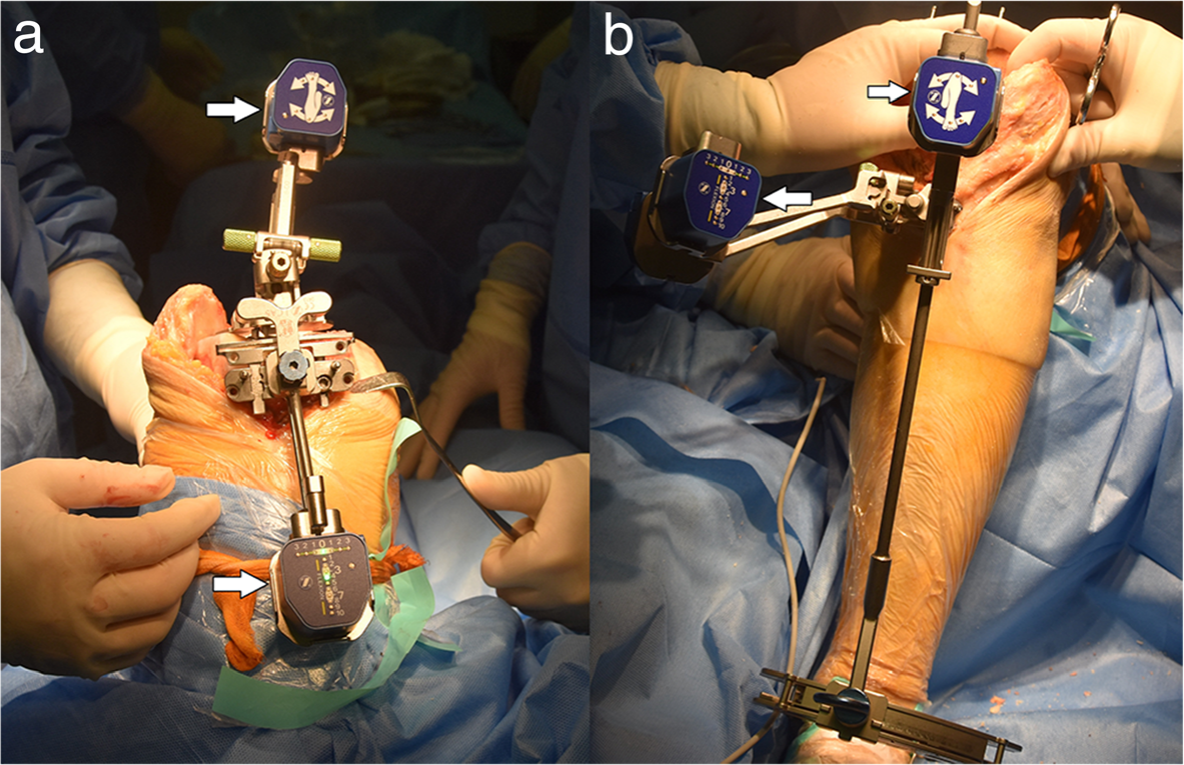
Intraoperative view of the iAssist instruments. **a** View of the femoral cutting block with the pods (white arrow) for distal femoral cut. **b** View of the extramedullary tibial cutting block with the pods (white arrow) for tibial cut. A LED light on pods is used as an indicator for proper cutting angle in a real-time manner

Starting with the femur, a small spike was inserted into the distal femur using a pod clipped to the spike. Femur registration was performed using 13 stable positions by accelerating and stopping the leg to create a star-shaped or circular pattern and obtain the mechanical axis of the femur. Next, the femoral adjustment mechanism was installed with another pod clipped to it onto the anterior side of the femoral reference. Using gold and green screws, a light-emitting diode (LED) followed the pod to adjust the flexion/extension and varus/valgus independently (Fig. 1a). Then, the distal femur was resected using the distal femur resection instrument. A validation tool can be used to check the result or adjust the angle if necessary. Finally, a 4-in-1 cutting guide aligned in rotation on Whiteside’s line was used to finish the anterior and posterior femoral cuts. An EM tibial guide with a pod clipped to it was employed to obtain the mechanical axis of the tibia. The proximal tibial guide has two spikes. The longer spike was inserted into the proximal surface of the tibia between the two spines, and the claw of the distal tibial guide was positioned on the malleoli. The claw was confirmed to be at the centre of the ankle joint prior to the next step. Next, the tibia registration was completed through 3 stable positions: the abduction, adduction, and neutral positions. The tibial adjustment mechanism was used to adjust the flexion/extension and varus/valgus through the gold and green screws, respectively (Fig. 1b). The depth of the cut was determined using a classic stylus. A validation tool can be used to check the results or adjust the angle if necessary. In this study, there is one patient required additional resection of proximal tibia following validation. The soft-tissue release was carried out in 26 patients in this group.

### Surgical technique for the conventional group

The surgical procedure for the conventional group followed the standard TKA method. Starting with the femur, an intramedullary guide was employed to acquire the reference femoral mechanical axis. Next, an appropriate 4-in-1 cutting jig aligned in rotation on Whiteside’s line was used to finish the anterior and posterior femoral cuts. For the proximal tibia, an extramedullary (EM) cutting guide was used to acquire the reference of the tibial mechanical axis. The soft-tissue release was carried out in 25 patients in this group. The trial components were placed to examine the tension of the knee and the overall lower limb alignment. Then, the corresponding prosthesis was implanted.

The objective in both groups was to obtain femoral and tibial implants at 90° to the mechanical alignment and femoral rotation aligned with the transepicondylar axis and checked using Whiteside’s line. All surgeries in both groups were performed using the medial para-patellar approach with patellar eversion. The depth of the resectable distal femur was 9 mm, and the femoral valgus angle was set at 6°. The depth of the resectable proximal tibia was determined using a classic stylus depending on the size of the prosthesis (generally, the depth of the resectable bone outside of the proximal tibia is 9 mm). Depending on the actual condition, soft-tissue balance techniques were used to release the medial collateral ligament, lateral collateral ligament, or posterior cruciate ligament at different degrees to reach a balanced state (before releasing the soft tissue, redundant osteophytes must be eliminated). All patients underwent the same closure of wounds in a standard method and the same postoperative rehabilitation protocol. Anti-coagulants were stopped 1 week before the operation. The drainage tube was activated 4 h after surgery. Oral anti-coagulants were introduced to all patients on the second postoperative day.

### Radiological evaluation

**Fig. 2:**
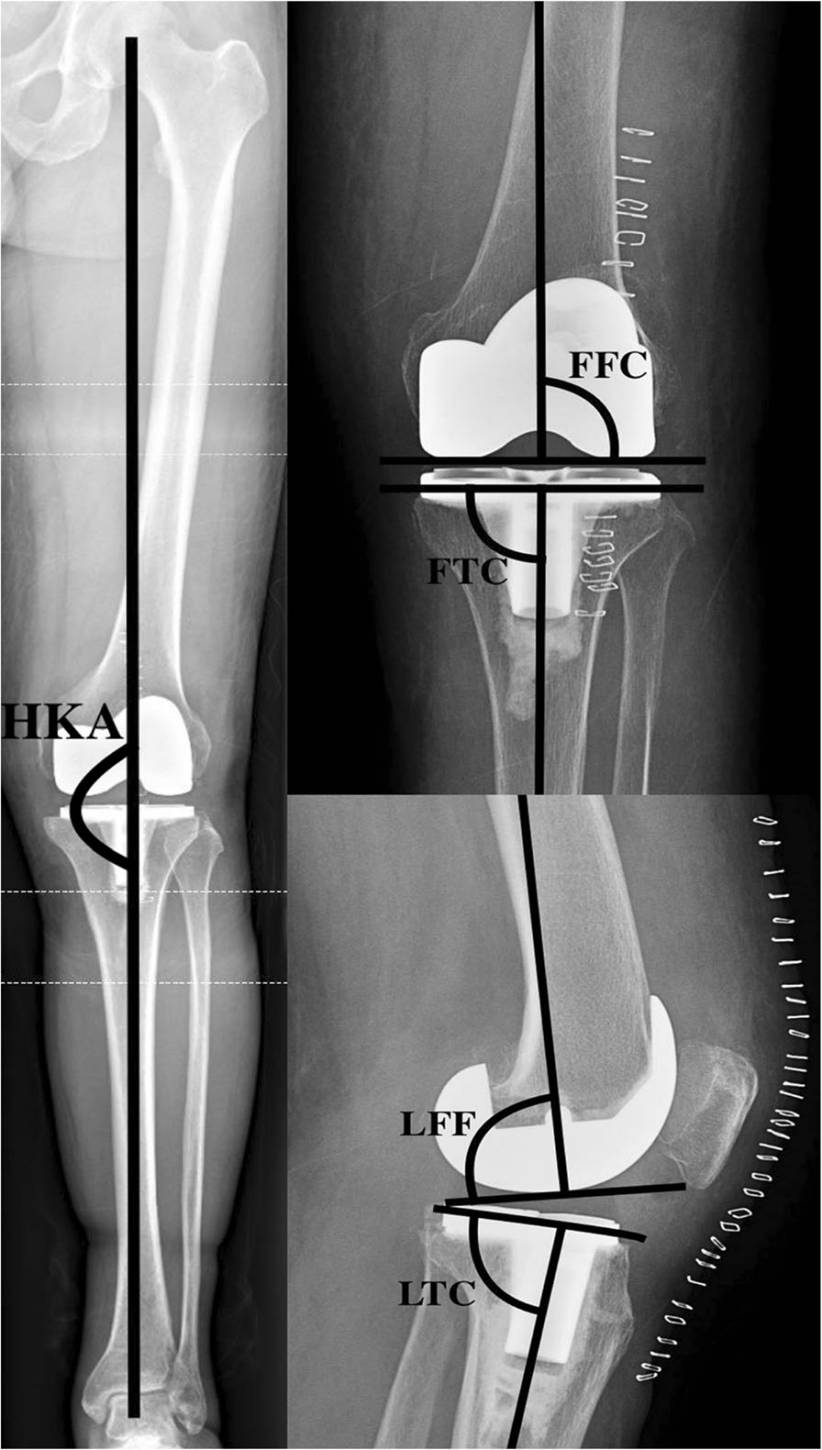
Antero/posterior and sagittal radiographs showing the standard graphic patterns of HKA, FFC, FTC, LFF, and LTC. HKA, hip-knee-ankle angle; FFC, frontal femoral component angle; FTC, frontal tibial component angle; LFF, lateral femoral flexion angle; LTC, lateral tibial component angle

A standardised radiographic assessment was performed to confirm the absence of malrotation in the radiographs (antero-posterior view, both the proximal tibia and fibula can be seen and are not overlying significantly; lateral view, the condyles of the femoral implant are overlying one another). X-rays were taken 1 week after surgery and included a total lower limb X-ray in a weight-bearing position, an antero-posterior view, and a lateral view. The assessor measured the HKA, frontal femoral component angle (FFC), frontal tibial component angle (FTC), lateral femoral flexion angle (LFF), and lateral tibial component angle (LTC) (Fig. 2). Calculations were completed to determine the absolute deviations of the HKA, FFC, FTC, LFF, and LTC from the ideal parameters (HKA, 180°; FFC, 90°; FTC, 90°; LFF, 87°; and LTC, 83°). The objectives used in this study were as follows: [1] restoration of an HKA value of 180° ± 3°, [2] satisfactory FFC and FTC defined as 90° ± 3°, [3] satisfactory LFF defined as 87° ± 3°, and [4] satisfactory LTC defined as 83° ± 3°. The radiographs were assessed twice by one experienced orthopaedic surgeon (CZH) who was not participated in surgery and blinded to the group to which the patient belonged using the IMPAX 6 (Agfa-Gevaert N.V., Mortsel, Belgium). To test intra-observer reliability, each set of measurements was repeated three times on 30 randomly selected patients. The intra-observer reliability based on ICC ranged from 0.878 to 0.901. All abovementioned parameters were compared between the two groups. The primary outcome of this evaluation was the proportion of radiological angle within 3° for HKA, FFC, FTC, LFF, and LTC, while the secondary outcomes include the absolute deviations of the HKA, FFC, FTC, LFF, and LTC.

### Short-term clinical evaluation

The gender, age, and BMI of the patients were recorded. Two experienced doctors handled the preoperative and postoperative clinical evaluations and were blinded to each other’s results. The Knee Society Score (KSS) and Western Ontario and McMaster Universities (WOMAC) scores were recorded at the final follow-up. The KSS involves 100 points each for a function (KSFS) and Knee Score (KSKS). The WOMAC contains 3 subscales (pain, stiffness, and physical function) consisting of 24 questions. Scores can range from 0 to 96 and higher scores indicate more severe disease. These two clinical outcomes, KSS and WOMAC scores, were assessed at the latest follow-up (mean follow-up period was 20.9 months in the CON group and 21.2 months in the ABN group). Haemoglobin (HGB) was recorded for all patients preoperatively and at postoperative days 1 and 3. The drop in HGB between the postoperative and preoperative days was calculated and compared between the two groups.

### Statistical methods

For all analyses, the independent variable was the patient group, and the dependent variables were the abovementioned parameters. A power analysis for HKA absolute deviation revealed that a sample size of 39 knees in each group was required to provide appropriate power (beta = 0.80, effect size = 0.5714286) based on previous literature [18]. To detect a minimal clinically important difference (MCID) in the KSS of 10 points from a baseline mean score of 80 with a standard deviation of 13, a sample size of at least 28 patients in each group would be required to achieve a power of 0.80 (effect size = 0.7692308) [18]. To detect an MCID of 11.5 points in WOMAC from a baseline score of 35 with a standard deviation of 10, a sample size of at least 18 patients in each group would be required to achieve a power of 0.95 (effect size = 1.15) [19]. There is currently no MCID specifically to measure HKA, FFC, FTC, LFF, LTC, and blood loss volume. These calculations were done for a one-sided test with a type I error of 0.05. Continuous data (age, BMI, HKA, FFC, FTC, LFF, LTC, blood loss volume, KSS, and WOMAC scores) are presented as the means ± standard deviations and compared using Student’s *t* test or the Mann-Whitney *U* test (non-normally distributed data). Categorical data (gender, the number of radiological angle within 3°) are presented as counts and percentages. Categorical data were compared using Fisher’s exact test. The postoperative HKA, FFC, FTC, LFF, LTC, blood loss volume, KSS, and WOMAC scores are the outcomes compared between the CON group and ABN group. All statistical analyses were performed by an independent investigator using SPSS (version 20.0; IBM SPSS Statistics, Chicago, IL, USA). Statistical significance was set at *P* < 0.05.

## Results

### Patient characteristics

A total of 204 patients were selected and divided into two groups (CON: 135, ABN: 69) after applying the inclusion and exclusion criteria. Then, the ABN and CON groups were propensity score matched in a 1:1 ratio based on age, gender, BMI, HKA angel, KSS, and WOMAC score. After propensity-matched analysis, 82 patients (82 knees) were finally included in this study who underwent TKA using conventional instruments (*n* = 41) or the ABN system (*n* = 41). Clinical results before and after propensity matching are summarised in Table 1. No significant differences were found in gender, age, BMI, or the preoperative HKA, KSS, or WOMAC score between the two groups after matching.

**Table 1:** Demographic characteristic of patients undergoing TKA with or without the use of ABN

	Unmatched group		SMD	*P* value	Propensity-matched group		SMD	*P* value
	CON	ABN			CON	ABN		
Number of cases	135	69			41	41		
Age (years)	66.4 ± 8.7	67.3 ± 7.6	0.847	0.347	67.7 ± 7.1	67.9 ± 7.3	0.021	0.826
Gender (female)	114	60	–	–	39	39		
BMI (kg/m^2^)	25.9 ± 2.7	27.3 ± 3.2	0.270	0.160	26.6 ± 1.5	26.8 ± 2.6	0.149	0.32
HKA (°)	174.4 ± 10.6	175.5 ± 10.2	0.106	0.491	175.1 ± 7.7	175.4 ± 8.4	0.038	0.711
KSS								
KS Knee Score	37.6 ± 17.1	40.8 ± 15.7	0.249	0.296	38.8 ± 14.2	39.6 ± 14.1	0.088	0.673
KS function Score	48.3 ± 17.8	51.4 ± 14.5	0.747	0.169	49.3 ± 8.7	50.6 ± 12.7	0.125	0.672
WOMAC score								
Pain	9.6 ± 4.3	8.7 ± 4.3	0.091	0.257	9.1 ± 3.9	8.9 ± 3.3	0.033	0.837
Stiffness	5.3 ± 1.7	4.9 ± 2.1	0.142	0.571	4.9 ± 1.5	4.8 ± 1.4	0.063	0.952
Function	40.7 ± 14.2	38.8 ± 15.1	0.237	0.771	39.2 ± 10.9	38.1 ± 11.1	0.051	0.923
Total	55.1 ± 11.4	51.8 ± 8.5	0.473	0.227	51.4 ± 9.2	50.9 ± 8.4	0.021	0.876
Follow-up (mouth)	23.7 ± 7.9	20.8 ± 6.7	–	0.327	20.9 ± 5.2	21.2 ± 5.3	–	0.609

*BMI* body mass index, *SMD* standardised mean difference

Continuous data are expressed as mean ± standard deviations

### Radiographic outcomes

There was a significant difference in the number of TKAs within 3° of HKA between the two groups (95.1% in the ABN group vs. 80.5% in the CON group, *P* = 0.043). In addition, the ABN group had significantly improved mean absolute deviations in the HKA (*P* = 0.033), along with the above-described trend towards increased accuracy. The numbers of FFC and LTC within 3° were significantly different (*P* = 0.021, *P* = 0.023, respectively) between the two groups. The ABN group also had significantly improved mean absolute deviations in the FFC (*P* = 0.004) and LTC (*P* = 0.031) compared to those of the CON group. There was no difference in the number of TKAs within 3° of FTC (*P* = 0.166) and LFF (*P* = 0.556), even significant differences in favour of iAsisst were found for mean absolute deviations in FTC (*P* = 0.017) and LFF (*P* = 0.023). These results suggest that a trend exists towards significant improvement with the use of the ABN system for mechanical alignment and component positioning (Table 2).

**Table 2:** Radiographic outcomes at final follow-up

	CON (*n* = 41)	ABN (*n* = 41)	*P* value
HKA absolute deviation	1.92 ± 1.5	1.51 ± 0.9	*0.033*
HKA within 3° achieved (*n*, %)	33, 80.5%	39, 95.1%	*0.043*
FFC absolute deviation	2.06 ± 0.88	1.21 ± 1.1	*0.004*
FFC within 3° achieved (*n*, %)	36, 87.8%	41, 100%	*0.021*
FTC absolute deviation	2.2 ± 1.4	1.3 ± 0.9	*0.017*
FTC within 3° achieved (*n*, %)	37, 90.2%	40, 97.6%	0.166
LFF absolute deviation	2.8 ± 1.3	1.7 ± 0.7	*0.023*
LFF within 3° achieved (*n*, %)	39, 95.1%	40, 97.6%	0.556
LTC absolute deviation	2.4 ± 1.7	1.4 ± 0.9	*0.031*
LTC within 3° achieved (*n*, %)	32, 78%	39, 95.1%	*0.023*

Absolute deviation: HKA, 180°; FFC/FTC, 90°; LFF, 87°; LTC, 83°. Continuous data are expressed as mean ± standard deviations. *P* values < 0.05 are in italics

*HKA* hip-knee-ankle angle, *FFC* frontal femoral component angle, *FTC* frontal tibial component angle, *LFF* lateral femoral flexion angle, *LTC* lateral tibial component angle

### Short-term clinical outcomes

At the final postoperative follow-up, the ABN group had a significantly higher KS function score (*P* = 0.032) (Table 3). However, no difference was found in the KS knee score (*P* = 0.767). At the final postoperative follow-up, the total WOMAC score was not significantly different between the two groups, but the pain and stiffness scores were significantly different (*P* = 0.034, *P* = 0.020, respectively). The mean reduction in HGB from preoperative to both postoperative days 1 and 3 was significantly lower in the ABN group than in the CON group.

**Table 3:** Clinical outcomes at final follow-up

	CON	ABN	*P* value
Knee Society Scores			
KS Knee Score	78.3 ± 17.1	76.4 ± 20.1	0.767
KS Function Score	70.7 ± 13.9	75.5 ± 15.2	*0.032*
WOMAC scores			
Pain	7.5 ± 2.3	6.3 ± 1.7	*0.034*
Stiffness	3.5 ± 1.3	2.7 ± 1.0	*0.020*
Function	24.5 ± 4.8	25.1 ± 6.6	0.778
Total	34.5 ± 4.5	32.7 ± 4.8	0.133
HGB drop 1d-post (g/L)	28.8 ± 4.1	19.3 ± 3.4	*< 0.001*
HGB drop 3d-post (g/L)	34.8 ± 3.1	20.3 ± 3.7	*< 0.001*

*HGB drop 1/3d-post*, the drop of haemoglobin at 1/3-day postoperatively

Continuous data are expressed as mean ± standard deviations. *P* values < 0.05 are in italics

## Discussion

Although the requirements of a neutral axis and optimal implant position have been challenged [19–21], a suboptimal implant position and alignment remain among the main reasons for TKA failure [10, 22, 23]. The Australian Orthopaedic Association National Joint Registry reported a reduced revision rate for navigated TKAs for loosening/lysis in patients aged < 65 years [24]. A set of studies have demonstrated that CAS has been shown to have greater accuracy than conventional instruments [25]. However, the limitations of CAS include large initial start-up costs, a substantial learning curve, an unstable workflow, femoral notching, large consoles, optical tracking, and line-of-sight problems. Moreover, the extra pin site may contribute to pain, infection, and pin-site fracture [14]. These complications will adversely affect TKA surgery, which is already a challenging invasive surgery. The aim of this study was to assess the accuracy of mechanical alignment, component positioning, and short-term clinical outcomes of TKA using a novel accelerometer-based navigation system to perform the proximal tibial and distal femoral resections when compared to conventional instruments.

Our study compared the accuracy of mechanical alignment and component positioning between CON and ABN groups. The results showed that there was a significant difference in the number of TKAs within 3° of HKA, FFC, and LTC between the two groups. However, we found no significant difference in the number of TKAs within 3° of FTC and LFF. In addition, the ABN group had significantly improved mean absolute deviations in HKA, FFC, FTC, LFF, and LTC when compared to the CON group. Our results confirm our initial hypothesis that compared with the use of conventional instruments, the use of the ABN for mechanical alignment and component positioning showed a trend towards significant improvement. Two recent meta-analysis studies found inconsistent results regarding the accuracy of ABN. Shigemura et al. reported that there were significantly fewer outliers for mechanical axis and coronal femoral component angle using ABN compare to CON group; however, no significant difference was observed for coronal tibial component angle outliers [16]. Sun et al. determined in their study that ABN was significantly superior to CON in reducing tibial component alignment out of ± 3°, femoral coronal angle out of ± 3°, and overall mechanical alignment out of ± 3°; and the two groups were comparable in tibial component posterior slope out of ± 3°, femoral sagittal angle out of ± 3° [17]. These results are partly consistent with our study.

In addition to the accuracy of mechanical alignment and component positioning, we also compared short-term clinical outcomes in terms of KSS, WOMAC, and blood loss between two groups. In this study, the ABN group had a better pain and stiffness score in WOMAC at the final follow-up. Our study also found that the KS function score was significantly better in the ABN group when compared to the CON group. Research shows that patients with increased symptoms of stiffness after TKA have a worse functional outcome and a lower rate of patient satisfaction [26]. Pain and stiffness are important factors related to the early functional rehabilitation and patient satisfaction. Postoperative pain after TKA plays an important role in delayed rehabilitation [27]. ABN system can reduce intraoperative injuries (with no need for an intramedullary location and less blood loss), which is a contributor to release pain after surgery and improve patient compliance during early rehabilitation. This may be one of the reasons of the better stiffness score in ABN group. However, our study found no significant difference in KS Knee Score and total WOMAC score between the two groups. This result is partly consistent with another study which is the only one assessed clinical outcomes of iAssist at 2-year follow-up and demonstrated no difference in KSS following TKA between ABN and CON groups [28]. No previous study has reported the WOMAC score in TKA using ABN. However, a high-quality meta-analysis study concluded that there is limited evidence which indicates that CAS improves functional outcomes at 5- to 8-year follow-up as measured by WOMAC and Knee Society Score-function scores [29].

Finally, we assessed blood loss using the reduction in HGB and found that the ABN system reduced hidden blood loss postoperatively, possibly because the ABN system did not need an intramedullary location or an extra pin site. Certainly, the concept fits with the research conducted by Ikawa et al. [30]. Diamond et al. found that higher HGB at baseline was associated with rehabilitation outcomes after TKA [31]. Although TKA is an effective, approved treatment for advanced arthritis of the knee, extensive bone resection, intramedullary positioning, and soft-tissue release during surgery often contribute to significant blood loss [15]. Despite recent advances in blood conservation, some patients require a transfusion after the operation, which can increase the risk of transfusion reactions and wound complications [32]. Reza et al. documented that compared with that of the conventional group, the CAS group can reduce transfusion risk [33].

Although the navigation system has been shown to reduce the risk of mal-alignment, whether the navigation system can improve the survival rate of the prosthesis and clinical outcomes for long-term follow-up is a question at issue. Several studies have documented a greater survival rate in a navigated group than in a conventional group 10 years after surgery [34, 35]. However, in some studies, no differences were found in long-term survival and the clinical outcomes between navigated TKA and conventional TKA [14, 20, 36, 37]. These discrepant reports may raise a concern that whether improved component and overall limb alignment accuracy guarantee improved clinical and functional outcomes for long-term follow-up. However, as summarised by Jones et al., by reviewing the long-term follow-up studies, the improved alignment accuracy achieved with navigation system may eventually translate to lower revision rates over time [38]. Based on clinical experience, precise mechanical alignment and implant positioning may significantly contribute to surgical success in patients with a high BMI, acute bowing, extra-articular deformity, or complex bone deformities in the long-term. Ritter et al. reported that compared with the failure rate of TKA in patients with a BMI of 23 to 26 kg/m^2^, the failure rate in patients with a BMI ≥ 41 kg/m^2^ increased from 0.7 to 2.6% (*P* = 0.0046) in well-aligned knees, from 1.6 to 2.9% (*P* = 0.0180) in varus knees, and from 1.0 to 7.1% (*P* = 0.0260) in valgus knees [10]. During the preparation of this study, one study reported that ABN system is accurate in achieving neutral mechanical alignment and optimal implant position after TKA in patients with extra-articular deformity [39].

There are several advantages of accelerometer-based navigation that should be noted here. The iAssist system has 4 pods that are clipped onto the cutting guide during surgery within the surgical field to facilitate direct observation of the cutting angle by surgeons. The surgical workflow of the iAssist system in TKA follows that of conventional instruments, thereby shortening the operation time and reducing the learning curve compared with those of CAS. The iAssist system exchanges information through a secure wireless local area network (LAN) to obtain a stable workflow. Compared with conventional navigation systems, the iAssist system is smaller and is not dependent on bone landmarks for location, which makes the operation easier [40]. Identifying the centre of the femoral head in TKA is difficult. The iAssist system provides a direct tracing method for the centre of the femoral head and allows accurate resection of the distal femur. Moreover, there are two literatures identified that iAssist system is as accurate as CAS [41, 42]. Moreover, the duration of surgery was significantly longer in the CAS group [42]. Due to the ABN system’s not needing of an intramedullary location for distal femur resection, it could be an appropriate tool for patients with femoral shaft disease or femoral implants [43].

Our study has several strengths. Propensity-based scoring helps reduce the bias inherent to observational studies, which can minimise possible confounding factors (age, gender, BMI, preoperative HKA, and clinical scores) [18]. All procedures in this study were performed by a single surgeon who was highly experienced with TKA and familiar with ABN technique prior to the commencement of this study (performed 30 navigated TKAs using ABN system before the first enrolled patient in this study), which can limit the risk of learning curve-induced bias. All patients were treated with standard postoperative rehabilitation protocols. The only difference was the way to guide bone resections between two groups. Although ABN system has been clinically applied for the past few years, the data published previously are still remaining few. The results from the present study could help to further understand the application of ABN system in TKA.

There were several limitations to this study. This study was not a prospective, randomised, and controlled comparison between ABN and CON. Due to the relatively new nature of ABN, this study could not determine the ABN system greatly contributed to long-term clinical outcomes and revision rates. These patients will need to be followed up annually to assess whether there are differences in clinical outcomes and revision rates between ABN and CON groups over the long term. Like most studies of alignment regarding TKA, the present study did not evaluate the rotational alignment. The cutting error between the intraoperative cut results and the postoperative radiographic alignment was not reported in this study. Relatively few cases of ABN were included in this study, though we controlled for this using PSM. Although this study was performed in a blinded manner, surgeon bias may have played a role. Furthermore, we did not compare the differences between the ABN system and other navigation systems.

## Conclusion

In summary, this study demonstrates that ABN system improved TKA mechanical alignment, component positioning, and decreased the hidden blood loss postoperatively compared to conventional instruments. However, no significant differences were found in clinical outcomes between ABN system and conventional instruments at the final follow-up (20.9 months in the CON group versus 21.2 months in the ABN group). Satisfying follow-up times are needed to confirm that the ABN system can contribute to TKA regarding the quality of life and prosthesis survival rate. ABN system should be treated reasoningly until the day that it shows definite improvements in clinical outcomes.

## Data Availability

The datasets used and/or analysed during the current study are available from the corresponding author on reasonable request.
